# Microglial phagocytosis induced by fibrillar β-amyloid is attenuated by oligomeric β-amyloid: implications for Alzheimer's disease

**DOI:** 10.1186/1750-1326-6-45

**Published:** 2011-06-30

**Authors:** Xiao-dong Pan, Yuan-gui Zhu, Nan Lin, Jing Zhang, Qin-yong Ye, Hua-pin Huang, Xiao-chun Chen

**Affiliations:** 1 Department of Neurology, Union Hospital of Fujian Medical University, 29 Xinquan Road, Fuzhou, 350001, China; 2 Fujian Institute of Geriatrics, Union Hospital of Fujian Medical University, 29 Xinquan Road, Fuzhou, 350001, China; 3 Centre of Neurobiology, Fujian Medical University, 88 Jiaotong Road, Fuzhou, 350001, China

## Abstract

**Background:**

Reactive microglia are associated with β-amyloid (Aβ) deposit and clearance in Alzhiemer's Disease (AD). Paradoxically, entocranial resident microglia fail to trigger an effective phagocytic response to clear Aβ deposits although they mainly exist in an "activated" state. Oligomeric Aβ (oAβ), a recent target in the pathogenesis of AD, can induce more potent neurotoxicity when compared with fibrillar Aβ (fAβ). However, the role of the different Aβ forms in microglial phagocytosis, induction of inflammation and oxidation, and subsequent regulation of phagocytic receptor system, remain unclear.

**Results:**

We demonstrated that Aβ(1-42) fibrils, not Aβ(1-42) oligomers, increased the microglial phagocytosis. Intriguingly, the pretreatment of microglia with oAβ(1-42) not only attenuated fAβ(1-42)-triggered classical phagocytic response to fluorescent microspheres but also significantly inhibited phagocytosis of fluorescent labeled fAβ(1-42). Compared with the fAβ(1-42) treatment, the oAβ(1-42) treatment resulted in a rapid and transient increase in interleukin 1β (IL-1β) level and produced higher levels of tumor necrosis factor-α (TNF-α), nitric oxide (NO), prostaglandin E_2 _(PGE_2_) and intracellular superoxide anion (SOA). The further results demonstrated that microglial phagocytosis was negatively correlated with inflammatory mediators in this process and that the capacity of phagocytosis in fAβ(1-42)-induced microglia was decreased by IL-1β, lippolysaccharide (LPS) and *tert*-butyl hydroperoxide (t-BHP). The decreased phagocytosis could be relieved by pyrrolidone dithiocarbamate (PDTC), a nuclear factor-κB (NF-κB) inhibitor, and N-acetyl-L-cysteine (NAC), a free radical scavenger. These results suggest that the oAβ-impaired phagocytosis is mediated through inflammation and oxidative stress-mediated mechanism in microglial cells. Furthermore, oAβ(1-42) stimulation reduced the mRNA expression of CD36, integrin β1 (Itgb1), and Ig receptor FcγRIII, and significantly increased that of formyl peptide receptor 2 (FPR2) and scavenger receptor class B1 (SRB1), compared with the basal level. Interestingly, the pre-stimulation with oAβ(1-42) or the inflammatory and oxidative milieu (IL-1β, LPS or t-BHP) significantly downregulated the fAβ(1-42)-induced mRNA over-expression of CD36, CD47 and Itgb1 receptors in microglial cells.

**Conclusion:**

These results imply that Aβ oligomers induce a potent inflammatory response and subsequently disturb microglial phagocytosis and clearance of Aβ fibrils, thereby contributing to an initial neurodegenerative characteristic of AD. Antiinflammatory and antioxidative therapies may indeed prove beneficial to delay the progression of AD.

## Background

Microglial phagocytosis has been proposed as an Aβ-lowering mechanism of Aβ immunization in Alzhiemer's Disease (AD) [1]. Microglia interact with fibrillar Aβ through the cell surface receptor system [2] that promote the clearance and phagocytosis of fAβ. The functional components of the receptor system include the scavenger receptor CD36, CD47 **(**integrin-associated protein), β1 integrin (Itgb1) [2-4], macrophage scavenger receptor class A (SRA) and class B (SRB) [5], receptor for advanced glycation end products (RAGE) [6,7], and the formyl peptide receptor (FPR) [8]. Exogenous microglial lateral ventricle transplantation has been shown to increase Aβ clearance in AD model rats [9]. Bone marrow-derived microglia can also efficiently restrict amyloid deposits [10]. These findings indicate the potential of exogenous and healthy microglia for therapeutic approach to AD. However, an enigma still remains: Why are those entocranial resident microglia surrounding plaques "activated" but unable to trigger an effective phagocytic response to engulf and degrade fibrillar Aβ deposits in ADż Recent evidence indicates that dysfunctional microglia is associated with aging [11,12]. Human brains containing high Aβ loads show a significantly higher degree of microglial dystrophy than nondemented, amyloid-free brains. Also, microglial cell senescence is exacerbated by amyloid [11,12]. Therefore, microglial degeneration may affect its phagocytosis and serve as an important factor in AD pathogenesis.

Abundant proinflammatory cytokines, chemokines, complement products, and oxygen radicals are presented in AD brains [13,14]. The binding of Aβ peptide to cell surface receptors induces proinflammatory gene expression and subsequently cytokines production [15]. Aβ seems to modulate these events all the time and interact with proinflammatory cytokines in a synergistic manner [16] to induce neuronal damage via reactive oxygen species (ROS)-dependent pathways [17]. ROS scavengers such as catalase obviously reduce the activation of nuclear factor kappa-B (NF-κB), a transcription factor mediating immune and inflammatory responses [18], and subsequently decrease the elevated Aβ-induced IL-1β level [19]. Accordingly, strategies to suppress oxidative stress and NF-κB activation may attenuate neuroinflammation and neuronal damage, which will be beneficial to AD treatment.

The processing of β-amyloid precursor protein (APP) by β- and γ-secretases produces Aβ peptides, of which Aβ(1-42) is especially biochemical active for its spontaneous proneness to oligomerization and fibrillation. Soluble Aβ oligomers rather than Aβ fibrils have been observed as the primary pathological species at early time points preceding fibril formation [20]. However, from APP processing to Aβ plaque formation, the specific role of Aβ oligomers and fibrils in mediating microglial activation is still unclear. Particularly, how do Aβ oligomers induce the generation of oxidative stress, inflammatory response and subsequently affect phagocytosis of Aβ fibrils in microgliaż Thus, the effect of Aβ components at different stages on microglia functions needs to be clarified so as to produce promising strategies to retard the early AD-related pathological affairs.

Here we investigated the differential effect of Aβ(1-42) oligomers versus fibrils on the viability of microglia, the expressions of inflammatory mediators, and phagocytosis function in microglia. Particularly, we applied the central Aβ components at the early and terminal stage (oligomers and fibrils, respectively) combined with some pharmacological agents to treat microglia in a proper sequential design, in order to study the role of Aβ components at different stages in microglial phagocytosis and cell surface components of the phagocytic receptor system, including CD36, CD47, integrin β1, SRA, SRB1, RAGE, FPR2, as well as the classical phagocytic receptors, the Ig receptors (FcγR I and FcγRIII). We further gained insights into the impact of anti-inflammation and anti-oxidation on oligomeric Aβ-activated microglial cells in NF-κB signaling.

## Results

### Effects of oligomeric versus fibrillar Aβ(1-42) on microglial cell viability

**Figure 1:**
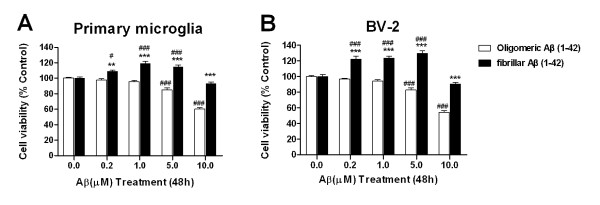
**Effects of oligomeric and fibrillar Aβ(1-42) on microglial cell viability**. Primary microglia and BV-2 cells were treated with oligomeric and fibrillar Aβ(1-42) (0~10 μM) for 48 h. The cell viability was assessed by MTT reduction assay. Values are expressed as mean ± S.E. (n = 5/group). ^#^*P *< 0.05, ^###^*P *< 0.001, compared with those of the control. **P < 0.01, ***P < 0.001, compared with those of oligomeric Aβ.

Previous studies have demonstrated that oligomeric Aβ(1-42) caused neuronal cells death *in vitro *[21-23]. However, the effect of oligomeric Aβ (oAβ) on microglial cell viability remains unclear. In this study, microglial cells were respectively treated with oAβ and fibrillar Aβ(1-42) (fAβ) at doses of 0.2 to 10.0 μM for 48 h. OAβ was shown to be cytotoxic to BV-2 and primary microglial cells when its dosage was up to 5.0 μM (Figure 1A). OAβ at a dose of 10.0 μM respectively resulted in a 45.7% and 39.8% reduction in the viability of BV-2 and primary microglial cells (*P *< 0.001, *vs *the control group). There were no significant changes in microglial cell survival when the dose of oAβ was within the range of 0.2~1.0 μM. In contrast, fAβ at doses of 0.2 to 5.0 μM resulted in a 22.0~29.4% increase in the viability of BV-2 cells (*P *< 0.001, *vs *the control group), and 8.8~19.3% in that of primary microglia (*P *< 0.05 or *P *< 0.001,,*vs *the control group) (Figure 1B), which suggests fAβ at lower doses well maintain the viability of microglial cells. However, fAβ at doses of 10.0 μM or higher did not affect the viability of primary microglia and BV-2 cells (*P *> 0.05, *vs *the control groups). These findings confirm that the non-neuronal toxicity of Aβ oligomes also occurs in microglial cells.

### Aβ(1-42) fibrils, not oligomers, enhanced phagocytic activity in a dose- and time-dependent manner in microglial cells

**Figure 2:**
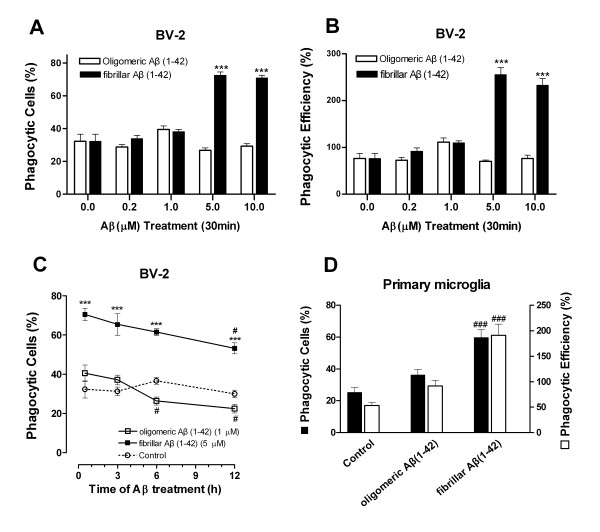
**Effects of oligomeric versus fibrillar Aβ(1-42) on phagocytic function of microglial cells**. (A) Dose dependence of phagocytosis stimulated by oAβ(1-42) and fAβ(1-42). BV-2 cells were incubated with the indicated Aβ(1-42) (0.2~10 μM) for 30 min before being exposed to microspheres for an additional 30 min; (B) The number of microspheres taken up per BV-2 cell with or without oAβ(1-42) or fAβ(1-42) treatment; (C) Time course of phagocytosis stimulated by oAβ (1-42) or fAβ(1-42) in microglial cell. BV-2 cells were preincubated with oAβ(1-42) (1.0 μM) or fAβ(1-42) (5.0 μM) for 30 min, 3 h, 6 h,12 h before the addition of microspheres for 30 min. (D) Phagocytosis stimulated by oAβ(1-42) or fAβ(1-42) in primary microglia. Cells were incubated with the oAβ(1-42) (1.0 μM) or fAβ(1-42) (5.0 μM) for 30 min before the addition of microspheres. Each value is expressed as mean ± S.E. of three independent experiments. ***P < 0.001, compared with the oAβ-treated groups. ^#^*P *< 0.05, compared with the 30 min treated groups.

Microglial phagocytosis was monitored by the ingestion of fluorescent microspheres. We investigated the dose and time course of the influence of oligomeric Aβ(1-42) and fibrillar Aβ(1-42) on phagocytosis. fAβ(1-42) enhanced microglial phagocytic function in a dose-dependent manner (Figure 2A, B). Exposure of BV-2 cells to fAβ(1-42) at a dose of 5.0 μM for 30 min produced the maximal phagocytic response, in which the percentage of phagocytosis cells and that of phagocytic efficiency were 72.6 ± 4.4% and 255.5 ± 29.9%, respectively. Those of the control group were 32.3 ± 8.7% and 76.4 ± 21.7%, respectively. However, oAβ(1-42) had no effect on microglial phagocytosis, even at a high treatment dose of 10.0 μM (Figure 2A, B), indicating that Aβ(1-42) fibrils specifically promotes microglial phagocytosis. Because oAβ (1-42) at the dose of 1.0 μM and fAβ(1-42) at the dose of 5.0 μM produced the maximal phagocytic response (Figure 2A, B) and they were not cytotoxic to microglial cells (Figure 1A, B), oAβ(1-42) at 1.0 μM and fAβ(1-42) at 5.0 μM were applied to the following time course study of microglial phagocytosis, respectively. As shown in Figure 2C, oAβ(1-42)-treated microglial cells demonstrated an initial, slight and transient phagocytic response to microspheres, which then declined over time (3~12 h) (*P *< 0.05 *vs *oAβ treated for 30 min). Interestingly, the elevated phagocytic response triggered by fAβ(1-42) occurred early after 30 min of incubation and kept at a high level for 3~6 h (*P *< 0.001, compared with the oAβ-treated groups) (Figure 2C) which slowly declined at 12 h over time (*P *< 0.05, fAβ treatment for 12 h *vs *for 30 min). Exposure of the primary microglia to Aβ(1-42) induced a phagocytic response of similar magnitude (Figure 2D). These results suggest that microglial cells exhibit a distinct phagocytic response to two conformations of Aβ(1-42).

### Differential impact of oligomeric versus fibrillar Aβ(1-42) on the expressions of inflammatory mediators in microglial cells

**Figure 3:**
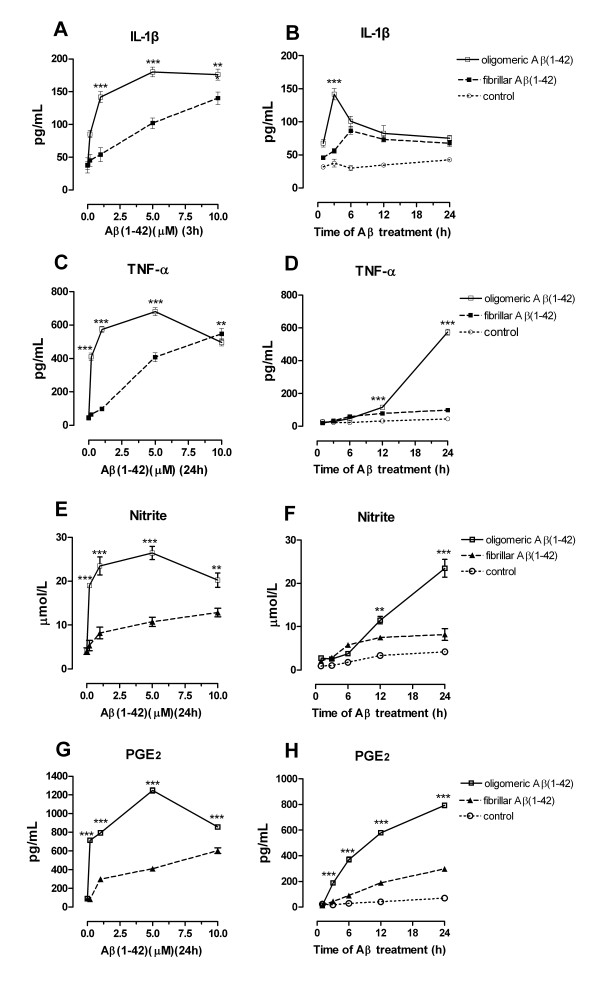
**Time course and dose response of inflammatory mediators induced by two types of Aβ(1-42) in microglial cells**. BV-2 cells were stimulated with two types of Aβ(1-42) (1.0 μM) for 1, 2, 3, 6, 12, 24 h. Or cells were stimulated with the Aβ(1-42) (0.2~10 μM) for indicated time (TNF-α, NO and PGE_2 _for 24 h, or IL-1β for 3 h), then the supernatant was collected for ELISA, EIA or Griess analysis, respectively. Values are shown as mean ± S.E. **P < 0.01 or ***P < 0.001, oAβ versus fAβ groups.

The current study also investigated the differential effects of oligomeric versus fibrillar Aβ (1-42) on IL-1β, TNF-α, NO, and PGE_2 _in microglial cells. OAβ(1-42) treatment resulted in a rapid and transient increase in IL-1β level (Figure 3B), while fAβ(1-42)-treated microglial cells released the inflammatory mediators in a slow and step-by-step manner (Figure 3D, F, H). IL-1β in oAβ-treated cells peaked at 3 h and declined from 3 through 24 h. In fAβ-treated cells, IL-1β peaked at 6 h, slowly declined and kept at a level from 12 through 24 h. These distinct expression patterns over time resulted in significantly higher IL-1β level in Aβ oligomers-treated cells than in fibrils-treated cells at the early stage (Figure 3A). Also, in the dose-dependent response study, oAβ treatment produced higher levels of TNF-α, NO, and PGE_2 _when compared with those of fAβ treatment (Figure 3C, E, G). In addition, the exposure of microglial cells to oAβ(1-42) produced the maximal response of TNF-α, PGE_2 _and nitrite release at 24 h, while the maximal response of IL-1β appeared at 3 h (Figure 3B, D, F, H), supporting the notion that IL-1β is a key proinflammatory cytokine at the early stage of glial activation. Exposure of the primary microglia to two conformations of Aβ(1-42) induced an inflammatory response of similar magnitude (data not shown). Taken together, these results prove the important role of Aβ oligomers in the proinflammatory response of microglia at the early AD stage, which is distinct from that of Aβ fibrils.

### Oligomeric Aβ(1-42) attenuated fAβ(1-42)-stimulated microglial phagocytosis

**Figure 4:**
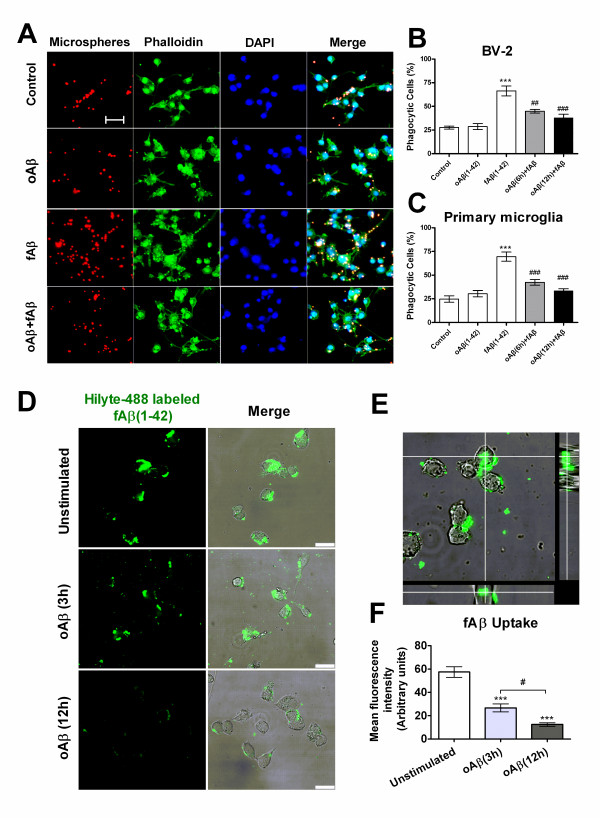
**oAβ(1-42) inhibits fAβ(1-42)-stimulated microglial phagocytosis**. BV-2 cells or primary microglia induced by oAβ(1-42) (1.0 μM), fAβ(1-42) (5.0 μM) alone or pretreatment of oAβ(1-42) for 6 h or 12 h followed by fAβ(1-42) treatment for 30 min. Fluorescent microspheres (red) were then added for 30 min. Cells were fixed with 4% PFA and stained with alexa488-conjugated phalloidin to visualize F-actin (green). The nucleus was visualized with DAPI (blue). (A) Fluorescent images of the distinct microglial phagocytosis. Scale bar = 40 μm. (B, C) Quantification of Aβ-mediated microglial phagocytosis. Data are presented as mean ± S.E. ***P < 0.001, compared with the control. ^##^*P *< 0.01, ^###^*P *< 0.001, compared with fAβ(1-42) alone. (D-F) Fluorescent labeled fAβ(1-42) internalization in microglia. Cells were pre-stimulated without or with oAβ(1-42) (1.0 μM) for 3 h or for 12 h, then incubated with Hilyte-488 labeled fAβ(1-42) (5.0 μM) for 30 min, and treated cells were fixed with 4% PFA and scanned under confocal microscope. Confocal images of microglial cells ingesting green Hilyte-488 labeled fAβ were shown in the *x-y *plane (D *left*). Overlays of phase-contrast and confocal images were shown in D *right*. Scale bars = 25 μm. (E) *z*-axis scans of cuts through the cells. (F) Quantification of internalized fluorescent fAβ in microglial cells. Values were expressed as mean fluorescence intensity (arbitrary units) of internalized fAβ per cell. Data are presented as mean ± S.E. ***P < 0.001, compared with the unstimulated control group. ^#^*P *< 0.05, between 3 h and 12 h of oAβ stimulation.

To mimic and investigate the roles of Aβ components at the early and terminal stages (oligomers and fibrils, respectively) in microglial phagocytic function, we used a design of a proper sequential-treated manner, that is, microglial cells were pre-stimulated with oAβ(1-42) (1.0 μM) for 6 h or 12 h, then followed by the addition of fAβ(1-42) (5.0 μM) before a 30 min incubation with microspheres. Intriguingly, the pretreatment of microglia with oligomeric Aβ(1-42) suppressed fAβ(1-42)-triggered phagocytosis. Microglial cells treated with fAβ(1-42) alone uptook more Nile red fluorescent microspheres (Figure 4A 1st column). For BV-2 cells, the percentage of phagocytic cells induced by the pretreatment of oAβ(1-42) for 6 h and 12 h followed by fAβ(1-42) treatment was ~21.5% and ~28.3% lower than that by fAβ(1-42) alone, respectively, (Figure 4B) (*P *< 0.001). Similarly, this effect occurred in primary microglia (Figure 4C). The percentage of phagocytic cells induced by the pretreatment of oAβ(1-42) for 6 h and 12 h followed by fAβ(1-42) treatment was ~27.1% and ~36.1% lower than that by fAβ(1-42) alone, respectively, (*P *< 0.001).

Since phagocytosis of fAβ is central to the role of activated microglia in AD, we further tested the effect of oAβ-prestimulated microglia on the phagocytic response to fAβ itself. oAβ(1-42)-stimulated and -unstimulated microglia with fluorescent labeled fAβ were incubated for 30 min, and cell-associated fluorescence intensity was measured (Figure 4D-F). Cells pre-stimulated with oAβ(1-42) (1.0 μM), compared with unstimulated cells, showed a respective decrease in uptaking of labeled fAβ (53.6% for 3 h and 78.2% for 12 h) (*P <*0.001) (Figure 4D, F). Moreover, the inhibition of oAβ(1-42) on microglial internalizating fAβ displayed a time-dependent effect (*P <*0.05) (Figure 4F). The simultaneous view of the entire cell (*x-y, x-z *and *x-z *plane) showed fluorescent labeled fAβ localizing in the cytoplasm. (Figure 4E)

These results suggest that the early stage component of Aβ, oligomers, can impair the microglial phagocytic function, and may subsequently impact the capacity of microglia to clear terminal fibrils Aβ or tissue debris in the brain.

### Oligomeric Aβ(1-42) attenuated fAβ-stimulated phagocytosis, which was correlated with the elevated inflammatory mediators

**Figure 5:**
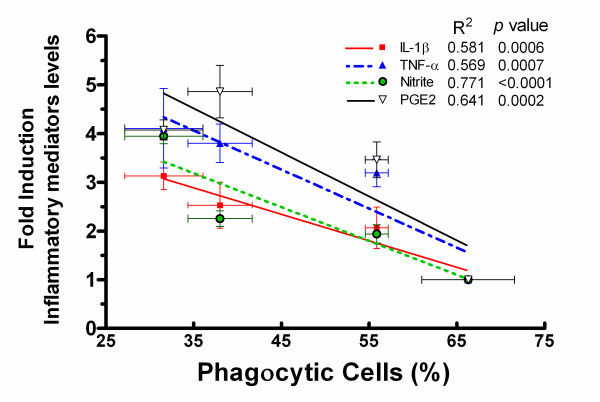
**Correlations between inflammatory mediators and the phagocytic response induced by oAβ in fAβ-stimulated microglial phagocytosis**. Primary microglia were pretreated with or without oAβ(1-42) (0.2, 1.0, 5.0 μM) for 12 h. The supernatant was collected to determine the levels of IL-1β, TNF-α, PGE_2 _and NO by ELISA, EIA and Griess analysis, respectively. On the phagocytosis assay, the cells were then treated with fAβ(1-42) (5.0 μM) for 30 min and incubated with fluorescent microspheres for another 30 min. Phagocytosis of the fluorescent microspheres was quantified as percent of phagocytic cells. Levels of IL-1β, TNF-α, PGE_2 _and NO were expressed as relative folds normalized to those of the control. Data are presented as the mean ± S.E. Pearson test was applied for the partial correlation analyses, *P *< 0.001.

Basing on the above observations, we further inquired whether the severe proinflammatory response triggered by Aβ oligomers was correlated with the suppressed phagocytic response mediated by fAβ in microglia and designed the following experiment. Microglia were pretreated with oAβ(1-42) at a range of concentrations (0.2~5.0 μM) for 12 h. Cultured supernatants were collected and assayed for inflammatory mediators including IL-1β, TNF-α, NO and PGE_2_. The bottomed cells were further treated with fibrillar Aβ(1-42) (5.0 μM) for 30 min and then incubated with fluoresent microsphere. *Pearson *test of correlation analysis revealed that there was a distinct negative correlation between the oAβ(1-42)-pretreated fAβ-stimulated microglial phagocytosis and the inflammatory mediators (R^2 ^= 0.58~0.64, *P *< 0.001) (Figure 5), indicating that there was a probable causality between the oAβ-suppressed fAβ-stimulated phagocytosis and the inflammatory response in microglia.

### Two types of Aβ(1-42) elicited the intracellular level of superoxide anion (SOA) in microglial cells: effects of NAC and PDTC

**Figure 6:**
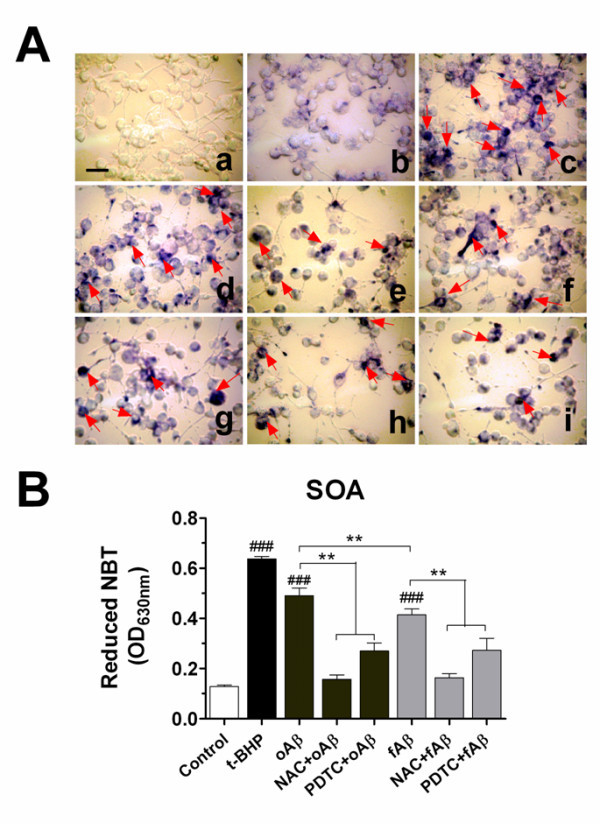
**Aβ induced the intracellular level of superoxide anion (SOA) in microglial cells: effects of NAC and PDTC**. Nitro blue tetrazolium (NBT) to formazan by superoxide anion (O_2_^-^) was used to measure the production of SOA. (A) BV-2 cells were pretreated with NAC (5 mM) or PDTC (20 μM) for 1 h and then stimulated with oligomeric Aβ(1-42) (1.0 μM) or fibrillar Aβ(1-42) (5.0 μM) for 12 h. Treatment of cells with t-BHP (100 μM) alone for 1 h was as a positive model control. Then NBT was incubated for a further 45 min and was fixed with methanol. The cells containing blue formazan particles (NBT-positive cells) were visualized under the microscope. (a): Blank (without NBT incubation); (b): Drug-free groups; (c): t-BHP alone; (d): oAβ alone; (e): NAC+oAβ; (f): PDTC+oAβ; (g): fAβ alone; (h): NAC+fAβ; (i): PDTC+fAβ. Scale bar = 50 μm. (B) NBT depositing inside the cells were dissolved with solubilization solution and the superoxide anion levels were quantified by the absorbance measured on a microplate reader at 630 nm. Each value is expressed as mean ± S.E. ^###^*P *< 0.001, compared with the drug-free group. **P < 0.01, compared between groups.

It has been reported that fibrillar Aβ(1-42) was able to trigger the production of superoxide anion-derived ROS in microglia [24]. We also tested whether oligomeric Aβ(1-42) elicited intracellular ROS generation in microglia. In an initial time-dependent response study, the treatment of BV-2 microglial cells with oAβ(1-42) produced the maximal response for SOA at 12 h (data not shown). Similar to a positive model treated with a strong oxidant, t-BHP (Figure 6A (c)), the treatment with oAβ(1-42) or fAβ(1-42) also indicated clear NBT positive cells (containing blue formazan particles) (Figure 6A (d, g)). NBT reduction quantified assay revealed that the production of SOA induced by oAβ (1-42) at 1.0 μM was ~20% higher than that by fAβ(1-42) even at 5.0 μM (Figure 6B) (*P *< 0.01). In addition, as expected, the treatment with pyrrolidone dithiocarbamate (PDTC), a nuclear factor-κB (NF-κB) inhibitor, or N-acetyl-L-cysteine (NAC), a free radical scavenger, markedly inhibited blue formazan particles formation and the production of SOA in BV-2 cells (Figure 6A (e, f, h, i) and 6*B*). The inhibitory efficiency of PDTC (20 μM) was 45%, whereas NAC (5.0 mM) resulted in a 68% reduction in the production of SOA in oAβ(1-42)-induced microglia. These results support that oxidative stress not only occurs in Aβ oligomers-induced microglia but is more intensive than that induced by Aβ fibrils, and that anti-inflammatory and anti-oxidative treatments may relieve this process.

### PDTC or NAC rescues oligomeric Aβ-elicited phagocytosis impairment in the inflammatory and oxidative milieu

**Figure 7:**
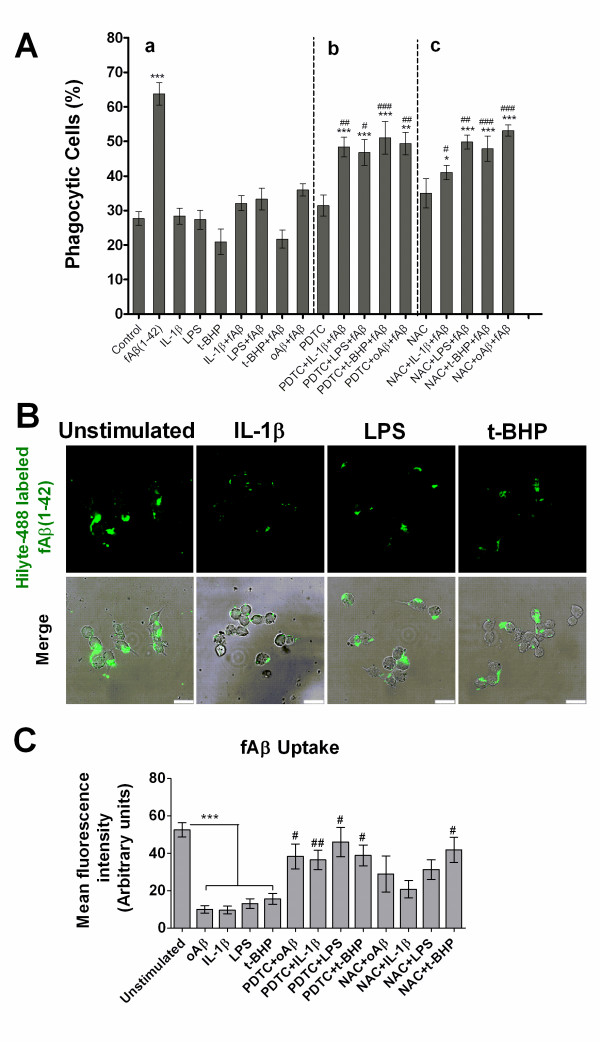
**Effects of IL-1β, LPS and t-BHP on the phagocytic response of microglial cells: regulation of PDTC and NAC**. BV-2 cells were pretreated with PDTC (20 μM) or NAC (5.0 mM) alone or with IL-1β (20 ng/ml) for 18 h or LPS (1.0 μg/ml) and oAβ(1-42) (1.0 μM) for 12 h or t-BHP (100 μM) for 1 h. On the day of phagocytosis assay, the cells were treated with fAβ(1-42) (5.0 μM) for 30 min and incubated with fluorescent microspheres for another 30 min (A) or incubated alone with Hilyte-488 labeled fAβ(1-42) (5.0 μM) (B-C). Phagocytosis of the microspheres was quantified as percent of phagocytic cells. (B) Confocal images of microglial cells ingesting green Hilyte-488 labeled fAβ. Overlays of phase-contrast and confocal images were also shown in D *right*. Scale bars = 25 μm. (C) Quantification of internalized fluorescent fAβ per cell. Values were expressed as mean fluorescence intensity (arbitrary units). Data are expressed as mean ± S.E. from the three independent experiments. *P < 0.05, **P < 0.01 or ***P < 0.001 compared with the unstimulated control; ^#^*P *< 0.05,^##^*P *< 0.01 compared with the drug (PDTC or NAC)-free group.

We further confirmed our hypothesis that oligomeric Aβ-elicited pro-inflammatory molecules and oxidative environments impair the capacity of microglial cells to phagocytose. Microglia were treated with IL-1β and LPS to induce a proinflammatory environment. IL-1β, LPS and an oxidant (t-BHP) were tested for their effects on fAβ-stimulated microglial phagocytosis and uptaking of fluorescent labeled fAβ. Also, anti-inflammatory agent and antioxidant, PDTC and NAC, were tested for their capacity to regulate microglial phagocytosis. As shown in Figure 7A-a, when microglial cells were exposed to IL-1β, LPS or t-BHP before fAβ stimulation, the capacity of microglial phagocytic response was distinctly suppressed (Figure 7A-a). The percentage of phagocytic cells pretreated with IL-1β, LPS, t-BHP or oligomeric Aβ decreased to near 50%, 48%, 66%, 44% of the induction by fAβ alone, respectively (Figure 7A-a). In contrast, the pretreatment of cells with PDTC or NAC relieved IL-1β, LPS, t-BHP or Aβ oligomers-impaired phagocytosis (Figure 7A-b, c). Phagocytosis changed only slightly in the presence or absence of pro-inflammatory revulsants alone (Figure 7A-a). Meanwhile, PDTC and NAC had no affect on microglial phagocytosis when added alone (Figure 7A-a).

Stimulation of microglial cells with IL-1β, LPS or t-BHP significantly reduced phagocytosis of fluorescence labeled fAβ to 18%, 26% and 30% of that of unstimulated cells, respectively (*P *< 0.001) (Figure 7B, C). Interestingly, as expected, the pretreatment of cells with PDTC markedly relieved IL-1β, LPS, t-BHP or Aβ oligomers-impaired phagocytosis (*P *< 0.05) (Figure 7C). NAC pretreatment also rescued t-BHP-impaired phagocytosis of fAβ (*P *< 0.05) (Figure 7C). Similar results were obtained with NAC counteracting IL-1β, LPS or Aβ oligomers-impaired phagocytosis, but differences were not statistically found (Figure 7C). Neither PDTC nor NAC treatment alone had affect on microglial phagocytosis (data not shown).

Collectively, these findings further support that Aβ oligomers-induced proinflammatory mediators and oxidative milieu negatively regulate microglial phagocytic function.

### Oligomeric Aβ, the inflammatory and oxidative milieu regulate gene expressions of Aβ-related cell surface receptors in microglial cells

**Figure 8:**
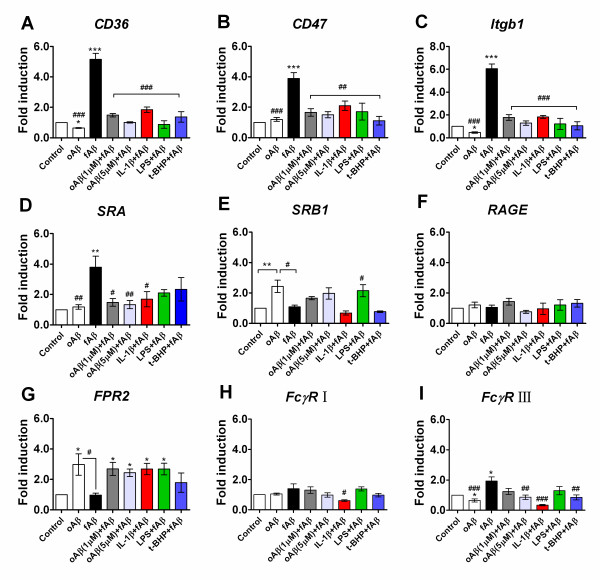
**Aβ-related cell surface receptors phenotype changes in microglial cells**. BV-2 cells were pre-stimulated with oAβ(1-42) (0, 1.0 and 5.0 μM) for 12 h or IL-1β (20 ng/ml) for 18 h or LPS (1.0 μg/ml) and or t-BHP (100 μM) for 1 h, then followed by an addition of incubation with fAβ(1-42) (5.0 μM) for 30 min. Total RNA was extracted, and mRNA expression of Aβ-related microglial cell surface receptors including *CD36, CD47, integrin β1 (Itgb1), SRA, SRB1, RAGE, FPR2, FcγR I and FcγRIII*, were analyzed by real-time PCR(A-I). All gene expressions were normalized to GAPDH level. The level of control expression was fixed at 1.0. Data were expressed as relative folds compared to control. Values correspond to mean ± S.E (n = 3). *P < 0.05, **P < 0.01 or ***P < 0.001 compared with the control; ^#^*P *< 0.05,^##^*P *< 0.01 or ^###^*P *< 0.001, compared with the fAβ(1-42) alone.

In order to explore why oAβ attenuated the fAβ-induced microglial phagocytic function, we investigated how oAβ affected specifically the Aβ-related microglial cell surface receptor system, including CD36, CD47, β1 integrin (Itgb1), SRA, SRB1, RAGE, FPR2, as well as the classical phagocytic receptors, the Ig receptors (FcRγI and FcγRIII). As shown in Figure 8, induction of microglial cells with fAβ (5.0 μM) for 30 min showed significantly increased mRNA expression of CD36 (4.2-fold increase; *P <*0.001), CD47 (2.9-fold increase; *P <*0.001), integrin β1 (Itgb1) (5.0-fold increase; *P <*0.001), SRA (2.8-fold increase; *P <*0.01), FcγRIII (1.0-fold increase; *P <*0.05), when compared with the basal level (Figure 8A-D, I), while fAβ did not change the mRNA expression of SRB1, RAGE, FPR2, FcγR I receptors in microglial cells. Interestingly, the pre-stimulation of oAβ (1.0 or 5.0 μM) for 12 h significantly downregulated the mRNA expression of CD36 (3.7-fold decrease for oAβ at 1.0 μM, 4.1-fold decrease for oAβ at 5.0 μM; *P <*0.001), CD47 (2.2-fold decrease for oAβ at 1.0 μM, 2.4-fold decrease for oAβ at 5.0 μM; *P <*0.001), Itgb1 (4.2-fold decrease for oAβ at 1.0 μM, 4.8-fold decrease for oAβ at 5.0 μM; *P <*0.001), as well as FcγR III (1.1-fold decrease for oAβ at 5.0 μM; *P <*0.01) in microglial cells, compared with fAβ alone induction (Figure 8A-D, I). Similarly, in the inflammatory and oxidative milieu, the pre-stimulation of microglial cells with IL-1β, LPS or t-BHP also decreased the fAβ-induced mRNA expression of CD36, CD47 and Itgb1 receptors (Figure 8A-C), in which the mRNA expressed change was 1.8- to 5.0-fold lower in the pre-stimulated group than that in the fAβ alone group (*P <*0.001). Moreover, IL-1β pre-stimulation decreased SRA (2.1-fold decrease; *P <*0.05), FcγR I (79% decrease; *P <*0.05) and FcγRIII (1.6-fold decrease; *P <*0.05) mRNA over-expression in fAβ-induced microglial cells (Figure 8D, H, I). In contrast, the stimulation of microglial cells with oAβ (5.0 μM) for 12 h resulted in a reduced mRNA expression of CD36 (36% decrease; *P <*0.05), Itgb1 (54% decrease; *P <*0.05), FcγRIII (35% decrease; *P <*0.05) (Figure 8A, C, I), but a significant increased mRNA expression of FPR2 (2.0-fold increase; *P <*0.05) and SRB1 (1.4-fold increase; *P <*0.01) (Figure 8E, G) compared with the basal level. However, oAβ stimulation did not change the mRNA expression levels of CD47, SRA, RAGE, FcγR I (*P *> 0.05) (Figure 8B, D, F, H).

These data indicate that microglia, when early exposed in the oligomeric Aβ or proinflammatory cytokines and oxidative milieu, have decreased the expression of Aβ-related cell surface receptors, and thereby may have decreased the capacity of microglia to bind and subsequently clear Aβ.

### Role of NF-κB signaling in the expression of inflammatory mediators in oAβ-stimulated microglial cells

**Figure 9:**
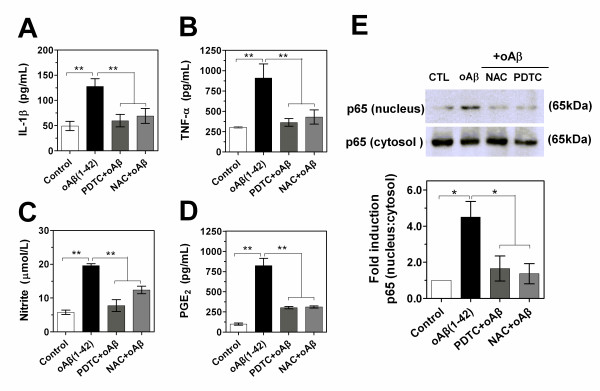
**Role of NF-κB signaling in the expression of inflammatory mediators in oAβ-stimulated microglial cells**. (A-D) BV-2 cells were incubated with oligomeric Aβ(1-42) (1.0 μM) in the presence or absence of NAC (5.0 mM) or PDTC (20 μM). After 24 h (TNF-α, NO and PGE_2_) or 3 h (IL-1β), the supernatant was collected for ELISA, EIA or Griess analysis. Values correspond to mean ± S.E. (n = 4). **P < 0.01. (E) Images and quantification data of Western blot showing NF-κBp65 protein. Whole cells and nuclear extracts were prepared after pretreatment of BV-2 cells with NAC (5.0 mM) or PDTC (20 μM) for 1 h before being stimulated with oAβ(1-42) (1.0 μM) for 1 h. Cytosol NF-κBp65 was used as a protein loading for nucleus p65. Ratio of nucleus p65 to cytocol represented the level of activation of NF-κB signaling. Representative of images revealed the effects of NAC and PDTC on the activition of NF-κB. Data were expressed as relative folds compared to control. Values correspond to mean ± S.E (n = 3). *P < 0.05, **P < 0.01.

NF-κB is an important upstream modulator of proinflamatory cytokine and iNOS expression. The involvement of NF-κB in PDTC and NAC-induced suppression of the production of cytokines, PGE_2 _and NO was further examined. As shown in Figure 9A-D, both PDTC and NAC dramatically decreased the oAβ-induced levels of TNF-α, IL-1β, PGE_2 _and nitrite. The ratio of nucleus to cytosol in NF-κB p65 protein, which reflected the level of NF-κB activation, was next examined by Western blot. In an initial time-dependent response study, the treatment of BV-2 cells with oAβ(1-42) produced the maximal response of NF-κB activation at the time-window of 30~60 min (data not shown). The rapid oAβ(1-42)-induced increase in the nuclear level of NF-κB p65 was dramatically blocked by PDTC and NAC (Figure 9E). Quantification of the protein revealed that PDTC and NAC reduced the ratio of nucleus to cytosol in NF-κB p65 protein by 63.2% and 69.6%, respectively (Figure 9E). These results suggest that NF-κB is an important moleculer target that determines the anti-inflammatory and anti-oxidative activity in oligomeric Aβ(1-42)-stimulated microglia.

## Discussion

The elucidation of the mechanism by which microglial phagocytosis is regulated, may help identify the etiology of Aβ desposit and therapeutic targets in aggregation-prone protein-associated neurodegenerative diseases such as Alzheimer's disease. The present study reports an important and exciting finding that extracellular oligomeric Aβ(1-42) suppresses the phagocytic function of microglia triggered by fibrillar Aβ. Our findings support the hypothesis that microglial dysregulation by oligomeric Aβ-elicited proinflammatory and oxidative stress milieu hampers clearance of fibrillar Aβ deposits, thereby leading to an initial neurodegenerative process characteristic of AD.

Oligomeric Aβ plays a critical role in the pathogenesis of AD. It is believed to contribute to early impairment of cognitive functions such as learning and memory [20,25]. In neuronal cells, it inhibits neuronal viability 10-fold more than fibrillar Aβ [21]. In microglial cells, the current findings reveal that oligomeric Aβ (≥ 5.0 μM) is more cytotoxic than fibrillar Aβ, suggesting that Aβ oligomers play a critical role in non-neuronal toxicity, whereby may lead to glial dysfunction.

Both pro-inflammatory cytokines and oxidative damage are observed early in the progression of AD [14,26] and can be detected prior to fibrillar Aβ deposition in AD brain [27]. Microglial activation can also be detected in vivo in around 50% of patients with mild cognitive impairment (MCI) [28], suggesting microglial activation is an early affair that involves progressive damage to immune system of AD patients. Our present data illustrate the phenotypic complexity of reactive microglia. Microglial cells treated with the two conformations of Aβ showed different profile changes of morphology and inflammatory mediators, including IL-1β, TNF-α, NO and PGE_2_. The Aβ-elicited microglial inflammatory responses were characterized by a conformation dependent manner. These results are in accordance with the works of Heuschling and colleagues [29,30]. They further demonstrated that the formylpeptide receptor 2 (FPR2) might mediate oAβ signaling and activate c-Jun and NF-κB pathway, which is also consistent with our current data that oAβ significantly upregulated the mRNA expression of FPR2, as well as our recent report [23] that Aβ oligomers can trigger a potent inflammatory response in microglia through NF-κB and JNK signaling. However, compared with their studies, our current data clearly displayed that the kinetics' profiles of time course and dose response of inflammatory mediators induced by the two forms of Aβ in microglial cells. Aβ oligomers resulted in a rapid and transient increase in IL-1β level. Compared with Aβ fibrils, they produced higher levels of TNF-α, NO, PGE_2 _and intracellular superoxide anion (SOA). In contrast, a higher concentration and a longer stimulating time were required for Aβ fibrils to induce microglial activation. Taken together, our present findings highlight the viewpoint that, at the early stage of AD, small diffusible oligomers activate microglia, leading to a more potent induction of inflammation, whereas fibrillar Aβ or plaques sustain the chronic inflammation at the terminal stage of AD pathogenesis. More importantly, our findings also reveal that the time course response of inflammatory mediators in microglia is correlated with the oAβ-impaired microglial phagocytosis stimulated by fibrillar Aβ.

Phagocytosis, a macrophage function critical for the uptake and degradation of infectious agents and senescent cells, contributes to the immune and inflammatory response and performs homeostatic activity in the normal CNS [31]. Microglia are competent phagocytes and are efficient in phagocytic uptake of amyloid aggregates [1,32,33] and senile plaques themselves [34] when examined in vitro. However, the limited clearance of dysfunction of microglia is characteristic of several neurodegenerative diseases [31]. In AD patients, the phagocytic function of peripheral blood mononuclear cells has been found to be impaired [35], suggesting that phagocytic function is also defective in AD.

The present study finds that an early stage component of Aβ, oligomers, is able to impair microglial phagocytic function and subsequently disturbs the capacity of terminal Aβ fibrils clearance. Its intriguing findings elucidate the mechanisms through which oAβ works: (1) oAβ stimulation downregulates the mRNA expressions of phagocytosing fAβ-related receptors such as CD36, integrin β1, as well as the classical phagocytic receptors, the Ig receptor (FcγRIII), whereas oAβ upregulates the expression of cell surface receptor genes (such as FPR2) which can induce a potent microglial proinflammatory response; (2) the prestimulation of oAβ or the inflammatory and oxidative milieu (IL-1β, LPS or t-BHP) significantly attenuates the fAβ-induced mRNA over-expression of CD36, CD47 and Itgb1 receptors. Therefore, these findings firstly provide a probable explanation for why "activated" microglia surrounding plaques lose their capacity to phagocytose Aβ deposits effectively during the terminal stage of AD brain. Furthermore, our observation also raises an intriguing question whether the initial microglial dysfunction induced by Aβ oligomers results in a decline in microglial-mediated clearance of tissue debris and microbes, e.g., viruses, bacteria, fungi, thereby, at the terminal stage of AD, increasing the risk of encephalic infectious disease, e.g., encephalitis.

It has been reported that microglia internalize oAβ through a nonsaturable, fluid phase macropinocytic mechanism that is distinct from receptor-mediated endocytosis [36], whereas microglia interact with fAβ through a characterized Aβ cell surface receptor complex comprising the B-class scavenger receptor CD36, α6β1 integrin, and CD47 [3]. In the present study, the fluorescent microsphere was used as a marker of fluid phase phagocytosis. We focused not only on the changes of microglial phagocytic function after the inducement with different Aβ forms (fAβ and oAβ) in a proper order, but also on the uptaking of fAβ itself by microglial cells. Our present data reveal that both oAβ and the inflammatory and oxidative milieu (IL-1β, LPS or t-BHP) significantly attenuated the fAβ-induced over-expression of FcγRIII gene and support the notion that for fluorescent microsphere itself, microglial phagocytosis, distinct from the internalization pathways of Aβ, may work through the mechanisms mediated by the classical phagocytic receptors, the Ig receptors (FcRγI and FcγRIII) or complement receptors [3].

The "inflammation hypothesis" stresses that hyperactive microglia are the primary cause of AD-associated neurotoxicity. In contrast, we propose that AD is caused not only by hyperactive but also by dysfunctional microglia. Microglial cells generate potentially damaging cytokines, nitric oxide, oxygen free radicals, and arachidonic acid derivatives, which could be mediators of the so-called secondary damage [37]. Dysfunctional microglia also show a significant reduction in the expression of their Aβ-binding receptors and Aβ-degrading enzymes, but maintain their ability to produce proinflammatory cytokines in AD [38,39]. These cytokines may in turn act in an autocrine manner and promote Aβ production by stimulating β- and γ-secretases and/or reduce Aβ clearance by reducing expression of Aβ-binding receptors and Aβ-degrading enzymes [38,39]. Therefore, together with our present results, we propose that, in a pathological condition like AD, oligomeric Aβ firstly triggers a rapid and potent inflammation and subsequently fibrillar Aβ sustains a chronic inflammatory environment, which suppresses the activation of phagocytic machinery, thereby affecting the ability of microglia to handle potentially toxic compounds, inhibiting clearance of fAβ and plaques, inducing a secondary immune response, and in turn aggravating brain inflammation. Antiinflammatory and antioxidative therapy can restore the functions of microglia, promote their capacity to clear Aβ, and decrease the production proinflammatory mediators, which may indeed be very helpful to delay the progression of AD.

A previous study reports that Aβ(1-42) fibrillization is a controlling factor in potentiating phagocytosis [40], which is attenuated by proinflammatory cytokines [41], and anti-inflammatory mediators, e.g., IL-4 treated microglia, enhancing the uptake and degradation of Aβ (1-42) [38]. In this study, our results firstly reveal that microglial phagocytosis was negatively correlated with oligomeric Aβ-induced inflammatory mediators and ROS. IL-1β, LPS and t-BHP all decreased the phagocytosis of fAβ induced-microglia, which could be relieved by a nuclear factor-κB (NF-κB) inhibitor (PDTC), as well as a free radical scavenger (NAC), suggesting that impaired phagocytosis by oAβ is mediated through NF-κB signaling dependent-inflammation and oxidative stress mechanism in microglial cells. Thereby, our results support a model in which the induction of oligomeric Aβ in microglia promotes oxidative damage and autocrine proinflammatory cytokine, which contributes to glial dysregulation and suppresses activation of the phagocytic machinery at the early stage of AD.

ROS is critical for inflammatory gene expression, including iNOS, in glial cells [42]. Microglia as a robust source of ROS increase oxidative stress and contribute to their dysregulation in AD. In this study, the elevated phagocytic response triggered by fAβ(1-42) occurred early after initial 30 min of incubation and kept at a high level for 3~6 h, but slowly declined at 12 h over time, which also indicates that the elevated ROS induced by fAβ itself may counteract with or negatively regulate the effect of fAβ-elicited phagocytosis. In addition, the pretreatment of PDTC or NAC mostly blocked the relocation of NF-κB and production of proinflammatory cytokines, whereas the same treatment did not restore the full capacity of phagocytic activity, suggesting that there may be other pathways involved in the process. Together with our current results, therefore, it is likely that there is a functionally relevant crosstalk between those different inflammatory events, e.g., secretion of ROS, cytokines, and phagocytosis.

Our findings illustrate that the exposure of microglial cells to oAβ(1-42) produces the maximal response of TNF-α, PGE_2_, and nitrite release at 24 h but that of IL-1β at 3 h, which supports that IL-1β is an immediate-response molecule and key immunoregulator at an early AD stage [43-45]. And the microglial proinflammatory response in AD may begin before the appearance of plaques in response to oligomeric Aβ. The intervention to prevent microglia activation should commence long before the appearance of Aβ deposits.

## Conclusions

The present study demonstrates that Aβ oligomers induce a potent inflammatory response, and subsequently disturb microglial phagocytic function preceding Aβ fibrils formation at an early AD stage. This provides a strong support for a novel view that β-amyloid conformation as an important determinant factor encourages sequential and progressive damage to the brain's immune system at different stages of AD pathogenesis. The present study also supports that anti-inflammatory and anti-oxidative therapies may facilitate the recovery of phagocytosis, the clearance of tissue debris and microbes, and the removal of fibrillar Aβ, and eventually ameliorate the pathologies of AD brain.

## Materials and methods

### Reagents

Dulbecco's modified Eagel's medium (DMEM), DMEM-F12, Hanks' balanced salt solution (HBSS), and fetal bovine serum (FBS) were obtained from Gibco (Grand Island, NY); phenol red-free F12 medium from PromoCell (Heidelberg, Germany); and 3-(4, 5-dimethylthiazol-2-yl)-2, 5-diphenyl tetrazolium bromide (MTT), LPS (from Escherichia coli serotype O111: B4), *tert*-butyl hydroperoxide (t-BHP), N-Acetyl-L-cysteine (NAC), nitroblue tetrazolium (NBT), and hexafluoroisopropanal (HFIP) from Sigma-Aldrich (St. Louis, MO). Aβ(1-42) peptide was purchased from Quality Controlled Biochemicals, Inc. (QCB, Hopkinton, MA); Lyophilized HilyteFluor™488 labeled Aβ(1-42) peptide was provided by AnaSpec (Freemont, CA, USA). NF-κB inhibitor, pyrrolidone dithiocarbamate (PDTC) from Calbiochem (La Jolla, CA, USA); IL-1β from R & D Systems (Minneapolis, MN); and Nile red fluorospheres and AlexaFluor488-phalloidin from Molecular Probes (Eugene, OR). Primary rabbit ployclonal antibody to mouse NF-κB p65 was from Cell signaling (Berverly, MA); 4',6-diamidine-2'-phenylindole, dihydrochloride(DAPI), and Westernblot Chemiluminescent Detection System (LumiGLO system) from KPL (Gaithersburg, MD); TNF-α and IL-1β ELISA kits from BioSource (Camarillo, CA); PGE_2 _EIA kit from Cayman (Ann Arbor, MI); TriPure Isolation Reagent and FastStart Universal SYBR Green Master (ROX) from Roche Diagnostics GmbH (Mannheim, Germany); and RevertAid™ First Strand cDNA Synthesis Kit from Fermentas (Shenzhen, China).

### Cells culture

#### BV-2 cells culture

The immortalized Murine BV-2 microglial cells were maintained in Dulbucco's modified Eagle medium (DMEM) supplemented with 5% FBS, 100 units/ml penicillin, and 100 μg/ml streptomycin, and were kept at 37°C in humidified 5% CO_2_/95% air. The cells were passaged every three days when growing up to 75% confluence.

#### Primary microglia culture

Microglia were derived from postnatal day 1 (P1) mouse brains (C57BL/6). Briefly, meninges-free cortices from P1 mice were isolated and trypsinized. Cells were plated onto tissue culture plastic in DMEM-F12 with L-glutamine containing 10% FBS and fed every three days. After 14 d, the cultures were shaken vigorously (120 min; 260 r.p.m. on a rotary shaker) to remove microglia.

### Preparation of Aβ(1-42)

Aβ(1-42) was prepared as previously described [21,46]. Briefly, lyophilized Aβ(1-42) peptide was initially monomerized by dissolving it to a final concentration of 1 mM in 100% hexafluoroisopropanal (HFIP) and separated into liquots in sterile microcentrifuge tubes. Then HFIP was evaporated under vacuum in a SpeedVac, and the peptide film was stored dessicated at -20°C until use. For the oligomers assembly, the peptide film was resuspended in dimethylsulfoxide (DMSO) to 5 mM with water bash ultrasonic for 10 min, then diluted to a final concentration of 100 μM in phenol red-free F-12 media, and incubated at 4°C for 24 h. To induce fibril formation, Aβ(1-42) was resuspended in sterile MinQ H_2_O and incubated for 1 week at 37°C.

### Cell viability Assay--MTT assay

MTT is converted in living cells to formazan, which has a specific absorption maximum. Cells were treated with Aβ(1-42) for indicated time periods and were further incubated for 4 h after the culture medium was changed to a medium containing 0.5 mg/ml MTT. Then, they were added with solubilization solution (10% SDS, 5% isopropanol in 0.012 M HCl) and incubated at 37°C in humidified 5% CO_2_/95% air for overnight. The absorbance of the supernatant was measured at 570 nm on an automated microtiter plate reader. Data were expressed as the mean percentage of viable cell versus control.

### Phagocytosis assay

Microglial cells were collected and 1 × 10^5 ^cells were plated in 24-well plates overnight. The medium was changed to serum-free DMEM, and 3 h later, the cells were incubated in the presence or absence of the revulsants (IL-1β, LPS, oAβ(1-42) or t-BHP) with or without inhibitors (PDTC or NAC) for indicated time periods. The fluorescent microspheres, as a marker of fluid phase phagocytosis, were then added to the treated cells for 30 min after having been washed in PBS containing 0.1% BSA. Cells were then fixed with 4% paraformaldehyde, and three random fields of cells (>100 cells) were counted under an inverted fluorescent microscope.

Phagocytic efficiency was determined by referring to Koenigsknecht et al. [3]. Briefly, the phagocytic efficiency was based on a weighted average of ingested microspheres per cell. The number of cells containing microspheres, the number of microspheres per cell, and the total number of cells were counted respectively. Phagocytic efficiency(%) = (1 × X_1_+2 × X_2_+3 × X_3_....+n × X*_n_*)/the total number of cells × 100%. X*_n _*represents the number of cells containing *n *microspheres (*n *= 1, 2, 3, ..., up to a maximum of 6 points for more than 5 microspheres ingested per cell).

### Phalloidin staining

The treated cells were rinsed with PBS before being fixed in 4% paraformaldehyde (PFA) and washed again in PBS. The cells were then incubated at room temperature with 0.1% Triton X-100 buffer for 5 min and washed again in PBS. AlexaFluor488 phalloidin (1:50 diluted in PBS) was added to the coverslips and incubated at room temperature protected from light for 20 min. Finally, coverslips were then incubated with DAPI (1:1000) for double staining. The coverslips were mounted on glass slides. The association of AlexaFluor488-labeled phalloidin was viewed under an inverted fluorescent microscope.

### Fluorescence labeled fAβ(1-42) phagocytosis assay

HilyteFluor™488 labeled Aβ(1-42) was aggregated according to the above fibril-forming condition. Primary microglia or BV-2 cells were plated at a density of 1 × 10^5 ^cells/well of a 24-well plate, then were pre-stimulated without or with oAβ(1-42) (1.0 μM) for 3 h or 12 h, LPS (1.0 μg/ml) for 12 h, t-BHP (100 μM) for 1 h and IL-1β (20 ng/ml) for 18 h. Stimulated or unstimulated cells were incubated with Hilyte-488 labeled fAβ(1-42) for 30 min at 37°C. Cells were washed and fixed in 4% PFA. Evaluation of Hilyte-488 labeled fAβ(1-42) phagocytosis in microglia was performed using a confocal microscope (Leica TCS SP5). A photomultiplier module was used to combine confocal with phase-contrast images to provide simultaneous views of the fluorescent fAβ and the entire cell to distinguish between phagocytosed fluorescent fAβ and fAβ adhered to cell surface. We counted intracellular fluorescent fAβ in three different experiments and analyzed 50 cells for each experiment. Data acquirement and analysis were performed with Leica Microsystems software (LAS AF Lite Version:1.8.1 build 1390).

### Measurement of TNF-α, IL-1β, PGE_2 _and nitrite levels

Microglial cells were stimulated with oligomeric and fibrillar Aβ(1-42) (1.0 μM) for 1, 2, 3, 6, 12, and 24 h. Or cells were stimulated with that of Aβ(1-42) (0.2~10 μM) for indicated time (TNF-α, NO and PGE_2 _for 24 h, or IL-1β for 3 h). The supernatants were collected and stored at -80°C until assays for TNF-α, IL-1β and PGE_2 _were performed. TNF-α, IL-1β and PGE_2 _levels were detected by mouse TNF-α, IL-1β ELISA kits and PGE_2 _EIA kit according to the procedures provided by the manufacturers. Accumulated nitrite (NaNO_2_) accumulation in the medium was used as an indicator of NO production as previously described [47]. The isolated supernatants were mixed with an equal volume of Greiss reagent (1% sulfanilamide, 0.1% naphthylethylenediamine dihydrochloride, and 2% phosphoric acid) and incubated at room temperature for 15 min. NaNO_2 _was used to generate a standard curve, and nitrite production was determined by measuring optical density at 540 nm. In the above studies with drugs (including PDTC and NAC), care was taken to ensure that cell viability was not altered under the concentrations of inhibitors used.

### Modified NBT assay for superoxide anion (SOA)

A modified assay for the intracellular conversion of nitroblue tetrazolium (NBT) to formazan by superoxide anion (O_2_^-^) was used to measure the production of reactive oxygen species [48,49]. In brief, 0.1% NBT was added to the media at the end of the treatment periods. As negative controls, BV-2 microglial cells were pretreated with 5.0 mM NAC 1 h prior to oligomeric or fibrillar Aβ(1-42) treatment. As a positive control, BV-2 cells were treated with 100 μM t-BHP for 60 min [50,51]. After incubation for 45 min at 37°C, the treated cells were washed twice with warm PBS, then once with methanol, and air-dried. The NBT deposited inside the cells was then dissolved with 240 μl of 2 M potassium hydroxide (KOH) and 280 μl of dimethylsulfoxide (DMSO) with gentle shaking for 10 min at room temperature. The dissolved NBT solution was then transferred to a 96-well plate and absorbance was read on a microplate reader at 630 nm. Meanwhile, the cells were allowed to adhere to glass cover slips placed in a 6-well flat culture plate. After similar treatments, NBT incubation, washing and fixing with methanol were carried out. The cells containing blue formazan particles (NBT-positive cells) were pictured under a microscope.

### Quantitative real-time PCR

Total RNA was extracted from the treated mouse BV-2 microglial cells using a commercially available assay (TriPure Isolation Reagent, Roche) according to the manufacturer's protocol. First-strand cDNA was synthesized with the use of 1 μg of total RNA (RevertAid™ First Strand cDNA Synthesis Kit, Fermentas). The quantitative PCR was performed with Applied Biosystems 7500 Real-Time PCR System (Applied Biosystems, Foster City, CA, USA) using SYBR Green to detect the amplification products. Reactions were as follows: 50°C for 2 minutes, 95°C for 10 minutes, and then 40 cycles of 95°C for 15 seconds followed by 60°C for 1 minute. Relative quantification of mRNA expression was calculated by the comparative cycle threshold (Ct) method after the target genes levels were normalized to expression of GAPDH housekeeping control gene for each sample. The fold difference in gene expression between treated groups was calculated as follows: fold difference = 2^-ddCt^, where ddCt = (Ct-_target _- Ct-_GAPDH_)_treated sample _- (Ct-_target _- Ct-_GAPDH_)_control sample_. Designed primers sequences [see Additional file 1, Table S1] were as follows: GAPDH, CD36, CD47, integrin β1 (Itgb1), scavenger receptor A (SRA), scavenger receptor B1(SRB1), RAGE, FPR2, FcγR I and FcγRIII.

### Nuclear extract

The treated cells were first resuspended with cold hypotonic buffer A [10 mM HEPES (pH 7.9), 10 mM KCl, 0.1 mM EDTA, 0.1 mM EGTA, 1 mM dithiothreitol, and 0.5 mM PMSF], followed by vigorous vortex for 15 s before standing at 4°C for 10 min and incubated for an additional 5 min after addition of 10% Nonidet P-40. The cytoplasmic protein was contained in the supernatant following centrifugation (6,000 × *g*, 4°C, 10 min). The pelleted nuclei were resuspended in cold buffer B [20 mM HEPES (pH 7.9), 25% glycerol, 420 mM NaCl, 1.5 mM MgCl_2_, 1 mM EDTA, 1 mM EGTA, 1 mM dithiothreitol, and 1 mM PMSF] and incubated for 20 min on ice, and nuclear lysates were then centrifuged at 14,000 × *g *at 4°C for 5 min. Supernatants containing the solubilized nuclear proteins were stored at -80°C for NF-κB assay.

### Western blot

The treated cells were washed with ice-cold PBS and then were incubated for 20 min with lysis buffer containing 10 mM Tris-HCl (pH 7.4), 100 mM NaCl, 1 mM EDTA, 1 mM EGTA, 1 mM NaF, 20 mM Na_4_P_2_O_7_, 2 mM Na_3_VO_4_, 0.1% SDS, 0.5% (w/v) sodium deoxycholate, 1% Triton-X 100, 1 mM PMSF, 60 μg/ml aprotinin, 10 μg/ml leupeptin, and 1 μg/ml pepstatin. Then the cells lysates were centrifuged at 12000 × *g *for 10 min. Nuclear and cytoplasmic extracts were separated by 10% sodium dodecyl sulfate-polyacrylamide gel electrophoresis (SDS-PAGE) and transferred to PVDF membranes. The membranes were probed with NF-κB p65 subunits antibody (1:750) to determine the efficiency of nucleocytoplasmic separation. Quantification of the band density was determined by densitometric analysis.

### Statistical Analysis

Data were shown by the means ± S.E. of at least three independent experiments. Statistical differences between values were determined by ANOVA followed by *Tukey post hoc *test, the partial correlation analyses by *Pearson *test. Significance level was set at *P *< 0.05.

## Competing interests

The authors declare that they have no competing interests.

## Authors' contributions

XP designed experiments, conducted all the experiments and wrote the manuscript; YZ participated in the preparation of Aβ peptides, conducted part of the experiments and revised of the manuscript; XC designed the study and reviewed the manuscript; NL and JZ participated in the image and data analysis. QY and HH participated in reviewing the manuscript. All authors read and approved the final manuscript.

## Supplementary Material

Additional file 1 Primers used for real-time PCR Click here for file
