# Supplementary material for: Microglial phagocytosis induced by fibrillar β-amyloid is attenuated by oligomeric β-amyloid: implications for Alzheimer's disease
Source: Mol Neurodegener. 2011 Jun 30;6:45. doi: 10.1186/1750-1326-6-45 (PMC3149591; doi:10.1186/1750-1326-6-45)
Supplement: Additional file 1 — Primers used for real-time PCR [file 1750-1326-6-45-S1.DOC]

**Supplemental Table 1. Primers used for real-time** PCR

| **Murine Gene** | **Forward Primer (5'-3')** | **Reverse Primer (5'-3')** | **Accession #** |
| --- | --- | --- | --- |
| **GAPDH** | CAGTGGCAAAGTGGAGATTGTTG | CTCGCTCCTGGAAGATGGTGAT | NM_008084.2 |
| **CD36** | GAACCACTGCTTTCAAAAACTGG | TGCTGTTCTTTGCCACGTCA | NM_007643.4 |
| **CD47** | TTGCGAAGTGACAGAGTTATCC | ACCTCCTTTCTCCTCCTCGTAA | NM_010581.3 |
| **Integrin β1** | CTGATTGGCTGGAGGAATGTA | TGTCCATCATTGGGTAAAACAA | NM_010578.1 |
| **SRA** | ACATCACCAACGACCTCAGACT | AGTTTGTCCAGTAAGCCCTCTG | NM_031195.2 |
| **SRB1** | TTTGGAGTGGTAGTAAAAAGGGC | TGACATCAGGGACTCAGAGTAG | NM_016741.2 |
| **RAGE** | GTGCTGGTTCTTGCTCTATGG | TTCCTGTGTTCAGTTTCCATTC | NM_007425.2 |
| **FPR2** | ATTGTTGCTGTTTGCTATGGAC | CTGCTGTAAGGACTCGTAAAGG | NM_008039.2 |
| **FcγR I** | CGGATGGAAGAATAAACTGGTG | GGTAGATGCCGCTGTGACTC | NM_010186.5 |
| **FcγRIII** | TTCTCTATCCCAAAAGCCAACC | GAGGATGTAGTTGCTGGGTCTT | NM_010188.5 |
